# P-Glycoprotein/MDR1 Regulates Pokemon Gene Transcription Through p53 Expression in Human Breast Cancer Cells

**DOI:** 10.3390/ijms11093039

**Published:** 2010-08-27

**Authors:** Shengnan He, Feng Liu, Zhenhua Xie, Xuyu Zu, Wei Xu, Yuyang Jiang

**Affiliations:** 1 The Key Laboratory of Chemical Biology, Guangdong Province, Graduate School at Shenzhen, Tsinghua University, Shenzhen 518055, Guangdong, China; E-Mail: liu.feng@sz.tsinghua.edu.cn (F.L.); 2 School of Life Science and Biopharmaceutics, Shenyang Pharmaceutical University, Shenyang 110016, Liaoning, China; 3 School of Medicine, Tsinghua University, Beijing 100084, Beijing, China

**Keywords:** Pgp (MDR1), Pokemon, p53, breast cancer

## Abstract

P-glycoprotein (Pgp), encoded by the multidrug resistance 1 (MDR1) gene, is an efflux transporter and plays an important role in pharmacokinetics. In this study, we demonstrated that the pokemon promoter activity, the pokemon mRNA and protein expression can be significantly inhibited by Pgp. Chromatin immunoprecipitation assay showed that Pgp can bind the pokemon prompter to repress pokemon transcription activity. Furthermore, Pgp regulated pokemon transcription activity through expression of p53 as seen by use of p53 siRNA transfected MCF-7 cells or p53 mutated MDA-MB-231 cells. Moreover, p53 was detected to bind with Pgp *in vivo* using immunoprecipitation assay. Taken together, we conclude that Pgp can regulate the expression of pokemon through the presence of p53, suggesting that Pgp is a potent regulator and may offer an effective novel target for cancer therapy.

## 1. Introduction

Multidrug resistance (MDR) plays an important role in chemotherapy treatment, but it is still under investigation whether it is the main one. Over-expression of ATP-binding cassette transporter superfamily members, which function as pumps to extrude anticancer drugs from cancer cells, closely relate to drug resistance phenotype [1]. P-glycoprotein (Pgp) is a member of ATP-binding cassette transporters that is often over-expressed in drug-resistant cancer cells. It is a 170 kD protein and is encoded by the human MDR1 (ABCB1) gene [2]. Pgp is expressed in only a limited number of human tissues with barrier function, including epithelia of the liver, kidney, small and large intestine and capillary endothelial cells in brain, ovary, and testis [3]. In addition, MDR1 expression has also been found in many, but not all, tumors from the adenal gland, liver, colon, kidney [3,4], and in one-third of patients with acute myelogenous leukemia at the time of first diagnosis, and in more than 50% of patients at first relapse [3]. The function of Pgp as a pump to extrude anticancer drugs from cancer cells has proven the significant role of Pgp in drug pharmacokinetics, and data from mdr knockout transgenic mice also strongly support the role of MDR1 in drug absorption, disposition, elimination, and detoxification pathways [5,6]

Pokemon (encoded by the Zbtb7A gene) is a member of the POK (POZ and Krüppel) family, which consists of an NH2-terminal POZ/BTB domain and COOH-terminal Krüppel-type zinc fingers [7]. Pokemon is identified as a critical factor in oncogenesis. Mouse embryonic fibroblasts lacking Zbtb7 are completely refractory to oncogene-mediated cellular transformation. Conversely, Pokemon overexpression leads to overt oncogenic transformation both *in vitro* and *in vivo* in transgenic mice [8]. It was shown that the survival rate of patients with negative pokemon expression was significantly higher than that of those with positive pokemon expression [9]. Further data shows that siRNA targeting-silencing of pokemon inhibited the growth of human Hela cells *in vivo* [10]. Pokemon also functionally acts as a transcription factor, which represses the tumor suppressor ARF gene by binding to its promoter region, potentially leading indirectly to p53 inactivation [8]. In addition, pokemon can also regulate the Rb gene, another tumor suppressor gene important in cell cycle arrest, by binding to the FREs(four GC-rich promoter elements) and competing with SP1 [11]. Besides, pokemon can enhances NF-κB-mediated transcription through an interaction between the POZ-domain of FBI-1 and the RHD of NF-κB [12]. It was also shown to regulate transcription of other genes including cyclin A and E2F4 [13]. Recently, it has been proved that pokemon represses transcription of p21 via competition with p53 and SP1 [14].

The p53 tumor suppressor gene has been often found mutated; in more than 50% human cancers [15]. p53 is a transcription factor that can be activated by several different types of DNA damage, including double-strand breaks in DNA produced by γ-irradiation and the presence of DNA repair intermediates after ultraviolet irradiation or chemical damage to DNA [15]. The p53 pathway plays multiple roles in cells, including tumor suppression, cell cycle arrest, and is involved in the control of cell proliferation, apoptosis, and DNA repair [16,17]. While dispensable for viability, in response to genotoxic stress, p53 acts as an “emergency brake” inducing either arrest or apoptosis, protecting the genome from accumulating excess mutations. Consistent with this notion, cells lacking p53 showed to be more genetically unstable and thus more prone to tumors [18]. It has been well established that p53 can regulate the expression of genes involved in control of the cell cycle and cell death on activation by genotoxic or oncogenic stress [19]. p53 can activate the transcription of the proapoptotic genes PUMA, PMAIP, Bax, Fas, and others, with repression of the transcription of the survival genes Bcl-2, MAP4, BIRC5 (survivin), Mcl-1, IGF-1R, MYC, EIF4E, and PIK3CA [20]. It is also reported that p53 inactivation can up-regulate Pgp expression [21].

Pgp locates at the end of some pathways, and it seems unlikely that Pgp plays an important role in regulating transcriptors in cancer cells. However, our previous article reported that Pgp can influence the expression level of survivin [22], and that survivin expression is regulated by coexpression of pokemon [23]. However, the correlation between Pgp and pokemon is unclear. In the present study, we show that Pgp can down-regulate pokemon expression level through the presence of p53, indicating a function of Pgp in tumor therapy.

## 2. Results and Discussion

### 2.1. Results

#### 2.1.1. Pgp Modulates Pokemon Expression

**Figure 1:**
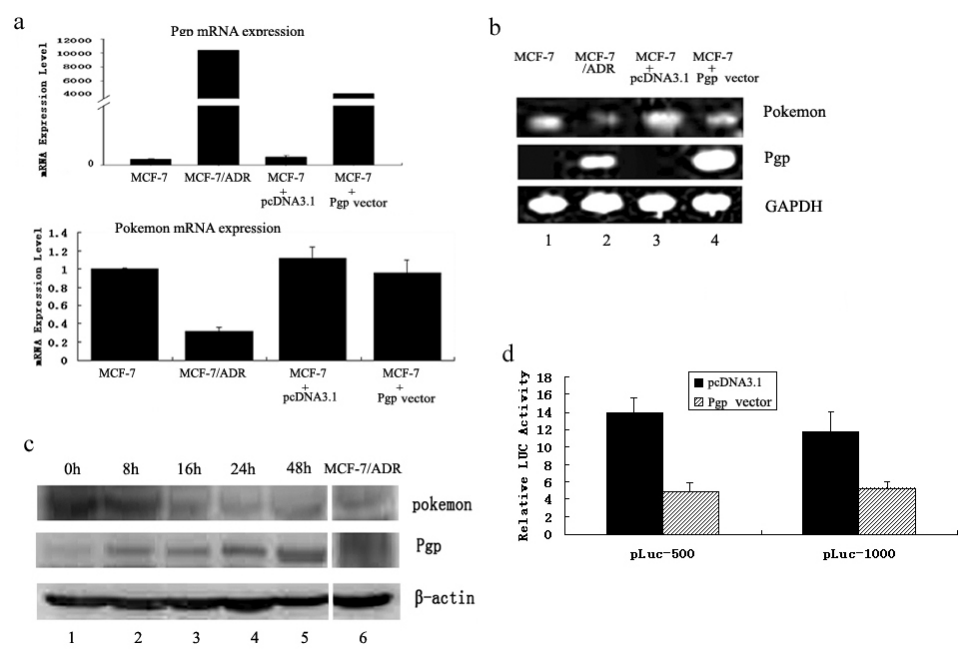
Transcriptional repression of pokemon by Pgp. (**a**) Modulation of pokemon mRNA expression by Pgp. (**b**) Comparison of pokemon and Pgp expression levels among MCF-7, MCF-7/ADR and MCF-7 cells transfected with pcDNA3.1and Pgp expression plasmid by RT-PCR analysis. (**c**) Expression of pokemon and Pgp was evaluated at the indicated times by Western blot. Similar results were obtained in three independent experiments (data not shown). (**d**) MCF-7 cells were cotransfected with Pgp expression vector and promoter-luciferase plasmids containing −1000 bp or −500 bp of pokemon promoter for 48 h. Results are presented as the stimulation of luciferase activity in the transfected cells relative to the luciferase activity in the cells transfected with the parental vector pcDNA3.1-Basic. Experiments were carried out in triplicate and a representative experiment is shown. Error bars represent the standard deviation of triplicate samples.

We initially found that in the MCF-7/ADR cell line, pokemon mRNA expression level was lower than that in MCF-7 cell line (Figure 1a, top and bottom). The most obvious difference between these two cell lines is the Pgp expression level, which is high in MCF-7/ADR cells (Figure 1a, bottom) while is not expressed in MCF-7 cells (Figure 1a, unit 1). Accordingly, we suspected that the inhibition of pokemon may be due to high Pgp expression levels. To test with, we performed a transfected assay. MCF-7 cells were transfected with a Pgp expression plasmid or the corresponding pcDNA3.1-Basic vector-alone as a control. We found that Pgp-positive breast cancer cells exhibited substantially lower pokemon transcript levels (Figure 1a, lane 3 and lane 4). RT-PCR showed the same result (Figure 1b). Next we detected whether the pokemon protein expression level was affected by Pgp. A time course of Pgp accumulation is shown in Figure 1c: a clear increase of Pgp protein levels in MCF-7 cells was observed within 48 h after Pgp transfection, and pokemon was down-regulated at the same time. No pokemon signal was detected in the Doxrubicin-resistant MCF-7/ADR cells (Figure 1c, lane 6), which would be consistent with the notion that pokemon expression is blocked by Pgp.

Next we tested whether the promoter activity of pokemon is restricted to Pgp. We previously demonstrated that the region −1000 bp immediately upstream of the transcription initiation site was the most active part on the pokemon promoter [24], so we used two promoter-luciferase plasmids containing −500 bp and −1000 bp fragments to examine whether the expression of Pgp could modify promoter activity of pokemon. Luciferase assays revealed that the region within −500 bp from the transcription site of pokemon is responsible for the induction by Pgp. As shown in Figure 1d, both of the promoter constructs cotransfected with Pgp significantly depressed luciferase expression compared with pcDNA3.1-Basic. The activity in the presence of these two fragments of pokemon promoter decreased by 65.2% and 55.2%, respectively. These results demonstrate that pokemon expression is regulated by Pgp.

#### 2.1.2. Mutation of p53 Affects the Repression of Pokemon by Pgp

**Figure 2:**
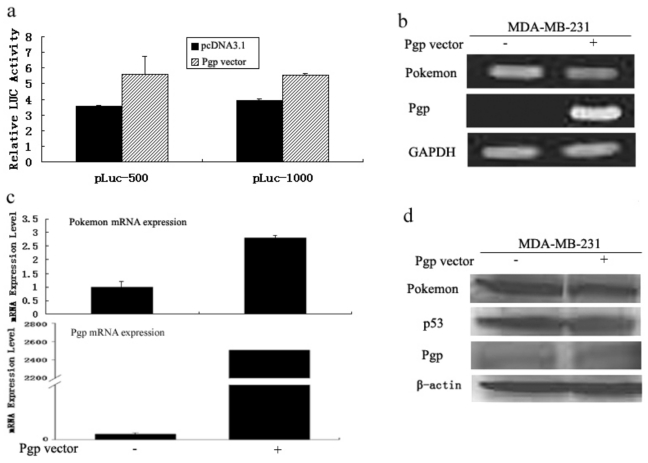
Mutation of p53 eliminates Pgp −mediated repression of pokemon. MDA-MB- 231 cells (p53 mutated) were used to investigate whether mutation of p53 affects the regulation of pokemon by Pgp. (**a**) Luciferase analysis of pokemon promotor activity in MDA-MB-231 cells transfected with pcDNA3.1 and Pgp expression vector. (**b** and **c**) Modulation of pokemon mRNA expression by Pgp in the presence of mutated p53. MDAMB- 231 cells were transfected with Pgp expression vectors. Pgp and pokemon mRNA levels were determined by RT-PCR (b) and real-time PCR analysis (c). (**d**) Immunoblot of pokemon, p53 and Pgp in MDA-MB-231 cells after transfection with pcDNA 3.1 or Pgp.

We used another breast cancer cell line MDA-MB-231 (p53 negative) to see whether the same phenomenon could be observed as in MCF-7. The luciferase assay was performed to identify whether the two promoters used above would be affected by Pgp in this cell line. After transfection of Pgp, we found that the activity from the pokemon promoter was induced instead of reduced (Figure 2a), most likely because of the loss of p53 activity. Next, we ectopically expressed MDR1 genes in MDA-MB- 231 and investigated the influence on endogenous pokemon expression. We suggested that the transrepression of pokemon was dependent on p53, because we did not detect any changes in the expression of this gene after transfected with Pgp in the p53 negative cell line (Figure 2b). In addition, the pokemon expression level increased after transfection with Pgp (Figure 2c) in MDA-MB-231 cells, as detected by real-time PCR, showing that down-regulation of pokemon is p53 dependent. Western blot analysis confirmed that the transcriptional repression of pokemon was mostly unleashed in p53 mutated MDA-MB-231cells (Figure 2d).

In general, we already demonstrated that Pgp could strongly reduce endogenous pokemon transcript levels (Figure 1). In contrast, pokemon expression was not affected by expression of Pgp in p53 negative MDA-MB-231 cell line. These findings indicate that suppression of pokemon by Pgp is mainly dependent on the tumor suppressor gene p53.

#### 2.1.3. The Degragation of Pokemon by Pgp Is p53-dependent

**Figure 3:**
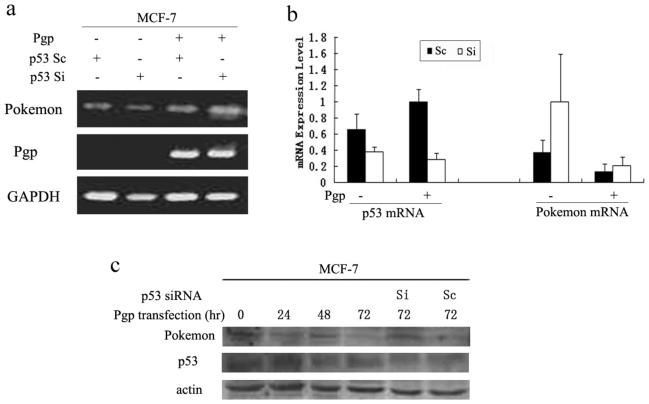
p53-dependent downregulation of the oncoprotein pokemon by Pgp. (**a**) RT-PCR analysis of MCF-7 cells transfected with Pgp or pcDNA3.1 and treated with siRNAs targeting p53 (Si) or scramble (Sc) gene expression for 48 h. GAPDH: internal standard. (**b**) mRNA levels of pokemon, p53 and Pgp in MCF-7 cells co-transfected with Pgp or vector and p53 siRNA or scramble RNA were detected by RT-PCR. (**c**) Levels of pokemon and p53 in MCF-7 cell extracts transfected with Pgp or vector in combination with p53 siRNA or scramble RNA were assessed by Western blot.

To further demonstrate that p53 is important for pokemon repression, MCF-7 cells were transfected with siRNAs, which partially blocked endogenous p53 expression level (Figure 3a,b). Therefore, we could assess possible alterations of pokemon expression at the mRNA level (Figure 3a,b). As shown in Figure 3a, treatment of MCF-7 cells with p53-targeting siRNAs led to a clear increase of pokemon transcription in the presence of Pgp. In contrast, pokemon expression was not upregulated by scramble siRNA, corroborating that the effect is p53-dependent. Next, a time course of MCF-7 cells after transfection with Pgp resulted in ~50% decrease of pokemon protein levels after 48 h and ~80% decrease 72 h after transfection (Figure 3c). To limit endogenous p53 expression, p53 siRNA was transfected and in this case repression of pokemon was rescued (Figure 3c, lane 5), whereas scramble siRNA had no such function (Figure 3c, lane 6). In summary, all the evidence above suggests that p53 could affect the repression of Pgp on pokemon expression to some extent. Thus, we come to the conclusion that p53 is required for the degradation of pokemon by Pgp.

#### 2.1.4. Pgp Combines with p53 and Interacts with the Pokemon Promoter *in Vivo*

**Figure 4:**
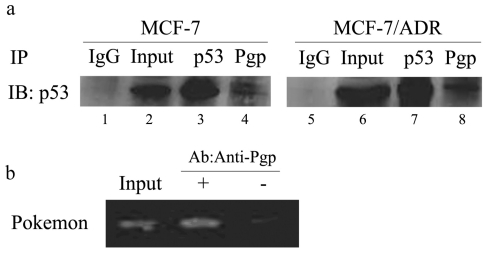
Pgp combine with p53 and interacts with the pokemon promoter *in vivo* in MCF-7 and MCF-7/ADR cells. (**a**) P53 protein is present in Pgp immunocomplexes in both MCF-7 transfected Pgp cells (lane 4) and MCF-7/ADR cells (lane 8). No p53 was immunoprecipitated with irrelevant antisera (rabbit IgG, lane1 5). Western blot was performed with a monoclonal antibody against p53 (DO-1, Sigma). (**b**) ChIP assay of the pokemon promoter in MCF-7/ADR cells. IP of formaldehyde-cross-linked lysate was performed using 0.3 μg anti-Pgp (Calbiochem), followed by PCR using oligocleotides specific for the pokemon promoter. As a positive control, lysate was incubated without antibody.

To address the possibility that Pgp utilizes p53 to repress pokemon transcription, immunocomplexes from MCF-7/ADR and MCF-7 cells transfected with Pgp were tested for the presence of a complex containing Pgp and p53. Immunoprecipitation (IP) of MCF-7/ADR extract with antisera specific to Pgp (C219, Calbiochem) consistently revealed the presence of p53 in the immunocomplex, whether endogenous (Figure 4a, lane 8) or exogenous (Figure 4a, lane 4), as ascertained by Western blot. The complexes persisted under stringent washing conditions (IP buffer washes, see Experimental section) and were not present when control antisera was used (rabbit IgG, lane 1 and lane 5).

It is known that Pgp is a protein which is expressed mostly on the membrane of cells. To investigate how it can influence the transcription factor in cell nucleus, we performed chromatin immunoprecipitation (ChIP) of the pokemon promoter using antisera specific for Pgp. In these experiments, Pgp showed a strong interaction with the pokemon promoter (Figure 4b, lane 2), whereas incubation with lysate without the Pgp antibody had no such strong binding. Therefore, we suggest that Pgp can affect pokemon through the recruitment to its promoter, and such recruitment may enhance binding p53 to the pokemon promoter to repress the transcription of pokemon.

### 2.2. Discussion

MDR is still an obstacle in the clinical management of breast cancer. Most of our knowledge on ABC transporters and their involvement in MDR is based on studies of Pgp, an organic cation pump that is the product of the ABCB1 (MDR1) gene. It is a full transporter comprised of 12 transmembreane segments divided into two transmembrane domains, each linked with an ATP-binding domain [25]. In cancer cells, Pgp is associated with the MDR phenotype, mediating resistance to anthracyclines, vinca alkaloids, colchicines, epipodophyllotoxins, and paclitaxel [26].

Previous reports have revealed that survivin expression is influenced by Pgp levels [22], suggesting that Pgp, the terminator of a pathway, affects factors upstream of it. Accordingly, our data demonstrated that Pgp could affect the oncogene pokemon through p53. This observation shows a possible link between Pgp and other transfactors. It provides strong evidence that there is a “feedback” relationship between effector and oncogene.

It is well-known that Pgp is a membrane protein that is mostly expressed at the cell membrane and several subcellular sites, including the nucleus [27], mitochondria [28,29], and Golgi apparatus [30]. However, we found that Pgp can act as a transcription factor to repress another gene in our experiments, and even get into the nucleus. We detected Pgp binding to the pokemon promoter in the MCF-7/ADR cell line, and interacting with the transcriptor p53 both endogenous and exogenous (Figure 4).

It is demonstrated that p53 is a tumor suppressor that is mutated in about 50% of human tumors [15]. Based on this, we speculated that p53 might play an important role in the regulation of pokemon. Indeed, from our experiments, the repression of pokemon by Pgp was found to mainly depend on p53 (Figures 2 and 3). It is possible that p53 was induced by Pgp and therefore it repressed the expression of pokemon. Here we propose two models for the mechanism of how Pgp affect p53: one is that Pgp has cleaved forms which can get into the nucleus, and the cleaved form could interact with p53; the second model is that a part of Pgp expressed on the inside of the nuclear membrane is responsible for the interaction with p53. In Western blot experiments detecting the expression levels of proteins, Pgp was observed as doublets, not as a single band, on nitrocellulose membranes (data not shown), suggesting Pgp has different forms *in vivo*. This supports the possibility mentioned above that Pgp may have cleaved forms, and some of them could act as transcription factors in the nucleus.

Recently, it has been reported that GATA-1 suppresses BIM-mediated apoptosis via pokemon, suggesting pokemon is a key downstream target of GATA1 [31]. Thus, we can conclude that pokemon is not only a transcription repressor that suppresses other genes’ expression, but is also controlled by some factors. Our data proved that it is indeed regulated by Pgp in the presence of p53.

## 3. Experimental Section

### 3.1. Cell Culture

MCF-7 cells were obtained from the American Type Culture Collection (ATCC, Manassas, VA, USA) and cultured in Dulbecco’s modified Eagle’s medium (DMEM). The MCF-7 ADR resistant cell line MCF-7/ADR (treated with Doxrubicin, which leads to Pgp overexpression in this cell line) was obtained from Nanjing Keygen Biotech. Co., Ltd. (China) and maintained in RPMI-1640 with 0.2 μg/mL ADR. The p53 mutated cell line, MDA-MB-231, was obtained from Cell bank, Shanghai Institutes for Biological Sciences, and propagated in Leibovitz L-15. All media were supplemented with 10% fetal bovine serum (FBS; Hyclone, Logan, UT, USA), and 100 units/ml of penicillin and 0.1 mg/ml streptomycin (Beyotime). Cells were cultured at 37 °C in a humidified atmosphere of 5% CO_2_ and 95% air.

### 3.2. Luciferase Assay

MCF-7 and MDA-MB-231 cells were plated at 20,000 cells per well in 24-well dishes and transfected 24 hours later with lipofectamine/DNA mixtures containing 1 μg total DNA which included 0.5 μg of luciferase reporter vector with promoter and 0.5 μg transcription factor. After 48 hours transfection, cells were harvested with lysis buffer and 10 μL of cell extract was mixed with 50 μL of luciferase assay buffer and another 10 μL was mixed with 50 μL of Renilla buffer [32]. The emitted luminescence was measured by Multimode Detector within 30 s.

### 3.3. RT-PCR

MCF-7 cells were plated in 24-well plates and transfected for 48 h with pcDNA3.1 or Pgp. Total RNA was isolated using TRIzol Reagent (Invitrogen) according to instructions provided by the manufacturer. The concentration of RNA was determined by UV spectrophotometry using a DU-800 Nucleic Acid/Protein Analyzer (Beckman Coulter). Reverse transcriptase (RT)-PCRs were performed using ReverTra Ace α (TOYOBO Life Science Department, Japan). Conditions for RT-PCRs were as recommended by the manufacturer. Glutaraldehyde-3-phosphate dehydrogenase (GAPDH) was used as an internal control. The corresponding 5’- and 3’-primers of pokemon, p53, MDR1 and GAPDH are in Table 1. The PCR cycling conditions are in Table 2. Five micro-liters of the RT-PCR mixture was used for agarose gel electrophoresis.

### 3.4. Real-Time PCR

The extraction of RNA and synthesis of cDNA are as described, and primers for p53, pokemon, MDR1, GAPDH are the same as used for RT-PCR. SYBR Green quantitative PCR amplifications were performed on the 7500 Real-Time PCR systems (Applied Biosystems). Reactions were carried out in a 20 μL volume containing 10 μL of 2 × SYBR Premix Ex Taq™ and 0.4 μL of 50 × ROX Reference Dye II. The thermal profile for the real-time PCR was 95 °C for 10 s followed by 40 cycles of 95 °C for 5 s, 60 °C for 34 s, followed the dissociation stage. The DCt data were collected automatically.

### 3.5. Western Blot Analysis

To determine the protein levels of Pgp and pokemon expression in MCF-7 cells, proteins were extracted according to conventional methods and separated by Sodium dodecyl sulfate-polyacrylamide gel electrophoresis (SDS-PAGE). After electrophoresis, the proteins were electrotransferred to nitrocellulose membranes, blocked and probed with anti-flag (1:1000), anti-Pgp (C219, 1:500), pokemon monoclonal (Sigma, 1:1000), anti-p53 (Sigma, 1:1000) or anti-actin (Beyotime, 1:1000) antibodies. The membranes were then incubated with horseradish peroxidase-conjugated goat antirabbit or anti-mouse secondary antibody, and detected with SuperSignal West Pico Chemiluminescent Substrate (Pierce, Thermo). The image was obtained using the Chemi Doc XRS imaging system (Bio-Rad).

### 3.6. ChIPs Assay

To analyze the physical interaction between Pgp and the pokemon promoter, MCF-7/ADR and MDA-MB-231 cells were used in ChIP assay. Cells were cross-linked by adding formaldehyde to a final concentration of 1%, and a final concentration of 125 mM glycine was used to stop the crosslinking. Cells were scraped and diluted in ChIP dilution buffer (1% Triton X-100, 0.1% Deoxycholate, 5 mM EDTA, 50 mM Tris at pH 8.1, and 150 mM NaCl) containing protease inhibitors. Chromatin was sonicated to an average length of about 500 bp while kept on ice. The sonicated supernatant was diluted with ChIP dilution buffer, and 20% of the total supernatant was saved as “input” at −20 °C while the rest was incubated with or without Pgp antibody (Calbiochem) on ice with rotation. The immune complexes were collected with Dynabeads protein G (Invitrogen). After washing with TE buffer pH 5.0 (10 mM Tris, 1 mM EDTA) three times, the pellet was dissolved in TE pH 3.0. DNA was purified using standard procedure with saturated NaCl, dimethyl carbinol and 70% ethanol. The primers for ChIP PCR are in Table 1. DNA was amplified as described in Table 2.

### 3.7. Immunoprecipitation (IP)

MCF-7 (transfected with Pgp), MCF-7/ADR and MDA-MB-231 cells were harvested and solubilized in 500 μL of IP buffer (20 mM Hepes, 0.2 mM EDTA, 5% glycerol, 150 mM NaCl, 1% Nonidet P-40) containing protease inhibitors (1 mM PMSF,0.1 mM DTT). Immunoprecipitation was performed using p53 antibody (Sigma, 1:1000), Pgp antibody (Calbiochem, 1:100) and IgG (Beyotime, 1:1000). Each IP was washed four times in IP buffer, then analyzed by Western blotting with p53 antibody (Sigma, 1:1000), followed by incubation with alkaline phosphatase-conjugated goat anti-mouse IgG (Beyotime, 1:2000) and visualized with SuperSignal West Pico Chemiluminescent Substrate (Pierce, Thermo).

## 4. Conclusions

Our results demonstrate that Pgp can regulate the expression of pokemon, and such regulation is through the presence of p53. The mechanism of how Pgp regulates Pokemon involves the combination of Pgp with p53 and recruitment to the Pokemon promoter. These findings identify Pgp as a regulator, based on its role in repression of pokemon, and provide further data on the relationship between oncogenes and tumor suppressor genes.

## Figures and Tables

**Table 1:** Oligonucleotide primers used in this study.

Name	Sequence	Name	Sequence
Pgp1	AAAGCGACTGAATGTTCAGTGG	p531	TGCGTGTGGAGTATTTGGATG
Pgp2	AATAGATGCCTTTCTGTGCCAG	p532	TGGTACAGTCAGAGCCAACCAG
Pokemon1	GAAGCCCTACGAGTGCAACATC	GAPDH1	GGTGGTCTCCTCTGACTTCAACA
Pokemon2	GTGGTTCTTCAGGTCGTAGTTGTG	GAPDH2	GTTGCTGTAGCCAAATTCGTTGT
ChIP1	GCCTGGCCAACATGGTGATAG		
ChIP2	ACGTGAAGGCGGTCAGATGTCG		

**Table 2:** RT-PCR and ChIP PCR conditions.

Pokemon			p53			Pgp			GAPDH			ChIP PCR		

Temp. (°C)	Time (min:sec)	Cycle	Temp. (°C)	Time (min:sec)	Cycle	Temp. (°C)	Time (min:sec)	Cycle	Temp. (°C)	Time (min:sec)	Cycle	Temp. (°C)	Time (min:sec)	Cycle
98	02:40	1	98	02:40	1	98	02:40	1	98	02:40	1	98	02:20	1
98	00:15	}26	98	00:15	}26	98	00:15	}26	98	00:15	}26	98	00:18	}27
60	01:00		60	01:00		60	01:00		60	01:00		60	00:30	
72	01:30		72	01:30		72	01:30		72	01:30		72	00:30	
72	06:00	1	72	06:00	1	72	06:00	1	72	06:00	1	72	10:00	1
